# *In*- and *ex-vivo* molecular imaging of apoptosis to assess sensitivity of non-small cell lung cancer to EGFR inhibitors using probe-based confocal laser endomicroscopy

**DOI:** 10.1371/journal.pone.0180576

**Published:** 2017-07-03

**Authors:** Florian Guisier, Pierre Bohn, Maxime Patout, Nicolas Piton, Insaf Farah, Pierre Vera, Luc Thiberville, Mathieu Salaün

**Affiliations:** 1 Department of Pulmonology, Thoracic Oncology and Respiratory Intensive Care & CIC INSERM U 1404, Rouen University Hospital, Rouen, France; 2 Normandie Univ, UNIROUEN, LITIS, Quant.I.F – EA 4108, Rouen, France; 3 Nuclear Medicine Department, Henri Becquerel Cancer Center and Rouen University Hospital, Rouen, France; 4 Cytology & Pathology Department, Rouen University Hospital, Rouen, France; University of South Alabama Mitchell Cancer Institute, UNITED STATES

## Abstract

**Background:**

Prediction of treatment outcome of non-small cell lung cancer (NSCLC) with EGFR inhibitors on the basis of the genetic analysis of the tumor can be incorrect in case of rare or complex mutations, bypass molecular activation pathways, or pharmacodynamic variations. The aim of this study was to develop an *ex vivo* and *in vivo* real-time quantitative imaging test for EGFR inhibitors sensitivity assessment.

**Methods:**

Erlotinib resistant (A549, H460, H1975), insensitive (H1650) and hypersensitive (HCC827) cell lines were injected subcutaneously in Nude mice. Tumor xenografts from mice treated with Erlotinib were imaged *ex vivo* and *in vivo* using probe-based confocal laser endomicroscopy (pCLE) and NucView 488 Caspase 3 substrate, a fluorescent probe specific for the activated caspase 3.

**Results:**

Assessment of apoptosis at 24h post treatment, both *ex vivo* in explanted tumor xenografts and *in vivo*, showed a significant difference between resistant cell lines (A549, H460 and H1975) and insensitive (H1650) or hypersensitive (HCC827) ones (p<0.05 for *ex vivo* imaging, p≤0.02 for *in vivo* imaging). There was also a significant difference between insensitive and hypersensitive cell lines, both *ex vivo* (p<0.05) and *in vivo* (p = 0.01).

**Conclusion:**

Real-time *in vivo* and *ex vivo* assessment of apoptosis using pCLE differentiates resistant from sensitive NSCLC xenografts to Erlotinib.

## Introduction

Over the past decade, identification of oncogenic molecular abnormalities in non-small-cell lung cancer (NSCLC), such as *Epidermal Growth Factor Receptor* (*EGFR*) activating mutations, has deeply changed the management of patients with advanced disease [1]. *EGFR* mutations convey constitutive activation of the EGFR and its downstream signaling pathways. Tumor cells bearing these mutations become highly dependent of the EGFR signal and thus are highly sensitive to EGFR tyrosine kinase inhibitors (EGFR-TKIs).

EGFR-TKIs have demonstrated a progression-free survival (PFS)[2–9] and overall survival (OS) [10] benefit in non-squamous NSCLC. Hence they have been implemented as standard first-line therapy for patients with metastatic NSCLC bearing activating *EGFR* mutations [11,12]. In patients with wild-type (WT) *EGFR* metastatic NSCLC, EGFR-TKIs may be used as second or third line treatment. The selection of patients on the basis of *EGFR* mutation analysis for first-line treatment with EGFR-TKIs has been successfully used in clinical trials, is now performed in routine clinical practice [13], and is considered the gold standard in Europe and in the U.S.. However, several issues remain regarding the relevant method for accurate prediction of EGFR-TKI sensitivity: (i) 15–30% of NSCLC bearing an activating *EGFR* mutation are insensitive to EGFR-TKIs in the clinical setting (2–10) (ii) a clinically relevant efficacy of EGFR-TKI is reported in another 10% of non squamous NSCLC without any *EGFR* mutation [14,15], (iii) *EGFR* mutation status may be unknown at the time of treatment initiation, (iv) a systematic *EGFR* testing of all NSCLC remains expensive and time-consuming.

In an effort to lower the cost of *EGFR* mutation screening, selection of patients on clinical, histological or biological criteria has been proposed and is widely used. The lower frequency of activating *EGFR* mutations among non-Asian, smoker or men and in squamous NSCLC, as well as the rarity of *KRAS* and *EGFR* double mutants may be used to exclude patients from such a screening [13]. To go further in that strategy, a score has been established to determine the probability of finding an activating *EGFR* mutation in a patient’s tumor [16]. All these strategies aim at predicting the sensitivity of the tumor cells to EGFR-TKIs. Another way to properly select the optimal treatment for patients could be the measurement of the biological effect of drugs on tumor cells. Specifically, the goal of such a strategy would be the set up of a rapid *in situ* test providing reliable information on how the tumor cells are affected by the drug.

It has been shown that early assessment of tumor response using 18-FDG PETscan is not predictive of patients’ outcome [17]. Other radio-tracers have been developed, which are specific of *EGFR* mutations [18], EGFR activity [19] or its downstream biological effect [20,21]. Notably, imaging of apoptosis has shown promising results [22–24].

Probe-based confocal laser endomicroscopy (pCLE) provides in-vivo, real-time and dynamic imaging of the distal lung areas during flexible bronchosopy [25–28]. Hence, pCLE offers the opportunity to observe biological processes at the cellular level in the lungs of patients, and has been used in human to establish the *in situ*, real time diagnostic of precancerous lesions [29], amiodarone-related pneumonitis [30], pulmonary alveolar proteinosis [31], and in animal models for pulmonary aspergillosis [32,33].

The NucView 488 Caspase-3 substrate (Biotium, San Francisco, California, USA), hereafter referred to as C3-NucView, is a fluorogenic dye that detects caspase 3 activity within intact cells, without interfering with the caspase 3 activity. The substrate is initially non-fluorescent, crosses the cell membrane to enter the cytoplasm, where it is cleaved by caspase 3 to release a high affinity DNA dye. The migration of the cleaved C3-NucView to the nucleus, and its interaction with DNA, leads to a nuclear bright green fluorescence, allowing caspase 3 activity detection.

We hypothesized that pCLE with C3-Nucview could be used *in-vivo* to image EGFR-TKI induced apoptosis in preclinical model and on *ex-vivo* fresh tumor samples at the microscopic level.

The objective of this study is to establish the feasibility of an Erlotinib sensitivity test using an *in situ*, real-time, quantitative imaging technique using pCLE with C3-NucView.

## Materials and methods

### Cell lines

Cell lines were chosen considering their sensitivity to Erlotinib: hypersensitive (HCC827, IC50 = 0.02μM), insensitive (H1650, IC50 = 2μM) and resistant (H1975, IC50 = 15μM; A549 and H460, IC50 = 20μM). All cell lines were kindly provided by Dr Richard Sesbouë (INSERM U1079, IRIB, Normandy University, Rouen, France). Cells were cultured in RPMI 1640 medium supplemented with 10% serum and 2mM Glutamine.

### Tumor xenografts

The study protocol was approved by the regional ethics committee for animal research (“Comité d’Ethique Normand en matière d’Expérimentation Animale”, agreement number N/01-06-12/11/06-15). Mice were bred in the animal facility of our institution, with unlimited access to food and water, and light/darkness cycle of 12h/12h.

For each tumor xenograft, cells were collected by trypsinisation and washed twice in PBS. 5.10^6 cells were resuspended in 100μL PBS and subcutaneously injected in the right flank of a 6 to 12-week-old female SWISS Nude mouse (Charles River, L’Abresles, France). Tumor engraftment led to visible tumors at 1 week and evaluable tumors (>125mm3) at 3 to 5 weeks.

### *In vitro* assessment of apoptosis using pCLE

For *in vitro* experiments, cell lines were treated with 10μM Erlotinib (AlfaAesar, Ward Hill, Massachusetts, USA), 30μg/mL Cisplatin (Mylan, Saint-Priest, France) or 0.2mL DiMethylSulfOxyde (Sigma Aldrich, Saint-Louis, Missouri, USA) for 18 hours. In order to demonstrate the specificity of the apoptotic signal using Nucview, cell lines were separated in two wells, in which were added ten μM Ac-DEVD-CHO (caspase 3 inhibitor) or DMSO for an additional 15 minutes. Cells were then harvested and a first sequence of images was acquired using the CellVizio^®^ system, by direct application of the optical miniprobe (Alveo-Flex AF2040, Mauna Kea Technologies) onto the cell pellets. Cells were re-suspended in 500μL of culture medium containing Erlotinib (10μM), Cisplatin (30μg/mL) or DMSO (0.2mL), and Ac-DEVD-CHO (10μM, Biotium) or DMSO. Ten minutes after addition of C3-NucView (0.2mM, Biotium), a second sequence of images was acquired using the same technique.

For flow cytometry experiments, cells were prepared and treated with Erlotinib (10μM), Cisplatin (30μg/mL) or DMSO (0.2mL), and Ac-DEVD-CHO (10μM, Biotium) or DMSO as described above. C3-NucView was added at 0.2mM. Cells were then analyzed on Cytomics FC 500 (Beckman Coulter, Fullerton, California, USA) within the hour. Five separate sets of experiments were performed.

### *Ex vivo* imaging of apoptosis

Once tumors have reached a 125mm^3^ volume, mice were anesthetized using isoflurane continuous inhalation, tumors were harvested and freshly divided into 4 to 8 2x2mm samples. Each sample was placed in 100μL medium, containing 10μL DMSO (n = 1), 10μg Cisplatin (n = 1), or 10μM Erlotinib (n = 2 to 6). After 10 minutes, all but one samples were transferred in 100μL medium containing 0.1nmol C3-NucView and 10μL DMSO (n = 1), 10μg Cisplatin (n = 1) or 10μM Erlotinib (n = 1 to 5). After an additional 2 minutes, samples were imaged as described above. Mice were then euthanized without anesthesia recovering, using intra-peritoneal injection of 20mg Pentothal.

### *In vivo* imaging of apoptosis

For in vivo preliminary experiments, each animal bared one A549 tumor in each flank, one being used as a control tumor. Once tumors reached a 125mm3 volume, intra-tumoral injection of Cisplatin 25μg in 0.05 ml saline or of 0.05mL saline was performed. After 24 hours, mice were anesthetized using isoflurane continuous inhalation. A 3mm skin incision was performed at the tumor site, a 24G needle was implanted into the tumor, and the 1.4mm Alveoflex miniprobe was inserted into the tumor through the needle. Different sets of images were acquired using CellVizio and the CellVizio software (version 1.2.3, Mauna Kea Technologies): prior to NucView injection and at 1, 15, 30, 60, 120, 180 and 360 minutes after intra-venous injection of 2nmol of C3-NucView. Mice were euthanized without anesthesia recovery.

For subsequent *in vivo* experiments, intra-peritoneal treatment was performed in animal groups bearing tumors from different cell lines. Once tumors have reached a 125mm^3^ volume, mice were treated with one intra-peritoneal injection of 25mg/kg Erlotinib or 0.1mL DMSO. After 24h, mice were anesthetized using isoflurane continuous inhalation, a 3mm skin incision was performed at the tumor site, a 24G needle was implanted into the tumor, and the 1.4mm Alveoflex miniprobe was inserted into the tumor through the needle. Two sets of images were acquired: prior to and 1 minute after the intra-venous injection of 2nmol of C3-NucView, using CellVizio and the CellVizio software (version 1.2.3, Mauna Kea Technologies). Mice were euthanized without anesthesia recovery.

### Western blot

In order to validate the specificity of the C3-NucView in our model, previously imaged A549 tumor xenografts from mice treated once with intra-tumoral injection of 25μg Cisplatin or 0.05mL saline were explanted at 24h post-treatment. Proteins were extracted, then loaded onto a 15% SDS-polyacrylamide gel, separated and transferred to a nitrocellulose membrane. The membrane was incubated with blocking solution at room temperature for 1h and incubated overnight with primary antibodies against caspase-3 (Cell Signaling Technology, Boston, MA, USA). After incubation with the corresponding HRP-conjugated secondary antibody (Santa Cruz Biotechnology), proteins were visualized using an enhanced chemiluminescence ECL Plus immunoblotting detection system (Amersham biosciences Europe GmbH, Freiburg, Germany).

### Fluorescence Intensity Ratio (FIR) establishment

Data were analyzed using the CellVizio Viewer 1.6.0 software (Mauna Kea Technologies). The software provides a fluorescence value in arbitrary unit (A.U) for each pixel of the image. A predefined 100 to 8000 Look Up Table (LUT) was applied to every image for comparison purposes. The 100 lower limit allows reduction of the background noise and excludes non fluorescent areas of the analysis. The upper limit of the LUT was set at its maximum, 8000 A.U, to avoid potential saturation of the signal. The ten brightest images of each sequence were selected on the basis of the median fluorescence intensity. For each experimental condition, the Fluorescence Intensity Ratio (FIR) was defined as follows: (sum of the 10 highest median fluorescence intensity in the sequence acquired with C3-NucView) / (sum of the 10 highest median autofluorescence intensity).

### Statistical analysis

Statistical analysis was performed using the R language and environment for statistical computing (version 3.1.3, R foundation for statistical computing, Vienna, Austria) on RStudio software (version 0.98.1103, RStudio^®^, Boston, MA, USA). Data were summarized using mean and standard error for mean. Giving the limited number of values, the non-parametric Kruskall and Wallis test was used for comparison of mean FIRs.

## Results

### *In vitro* imaging of apoptosis using pCLE

**Fig 1:**
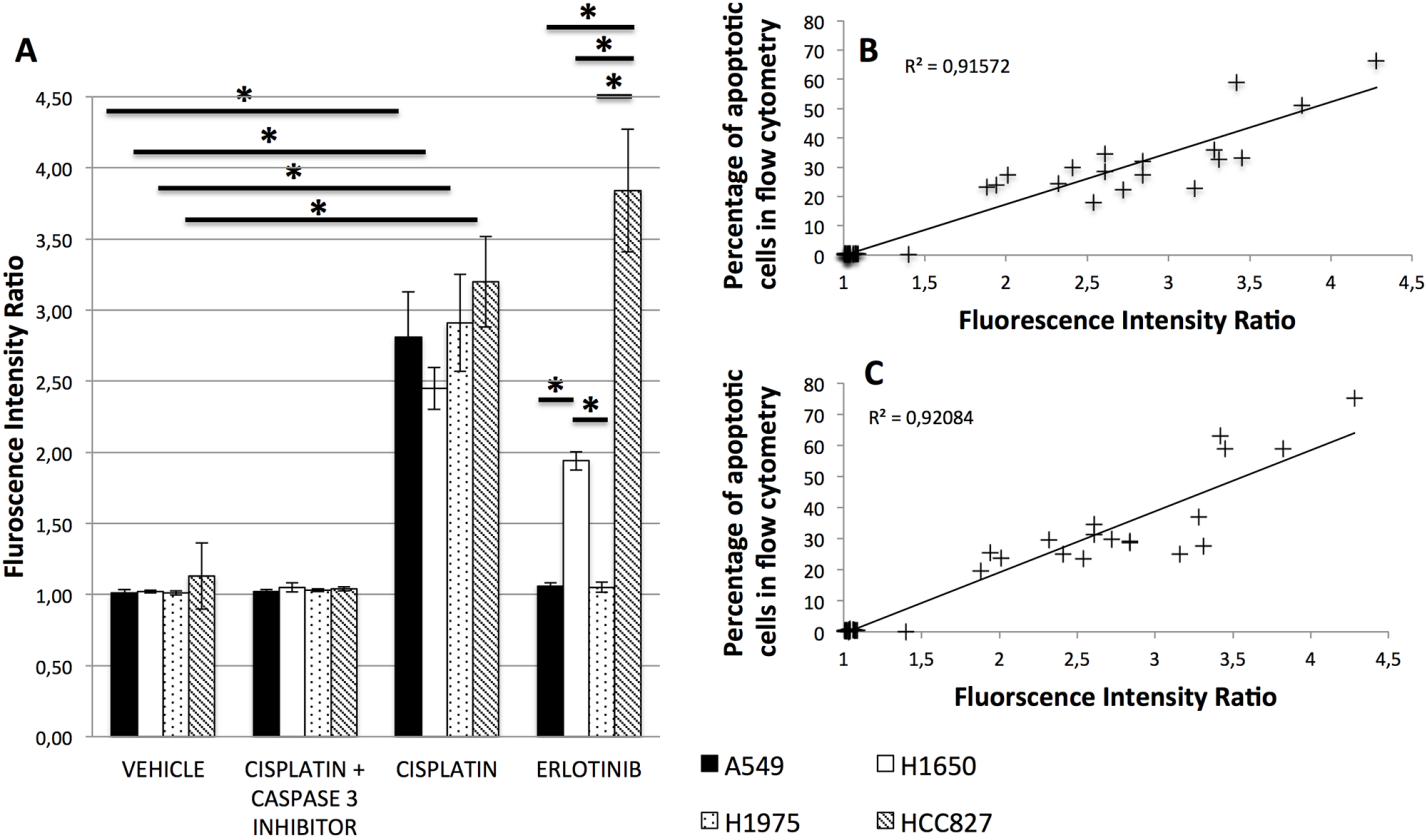
*In vitro* assessment of apoptosis using pCLE: The Fluorescence Intensity Ratio (FIR) is higher in sensitive cells, and is correlated to the presence of apoptotic cells. (A) A549, H1650, H1975 and HCC827 cell lines were treated for 18 hours with Erlotinib (10 μM), Cisplatin (30ug/mL) or DMSO (0.1%). Cells were then treated with ten μM Ac-DEVD-CHO (caspase 3 inhibitor) or DMSO for 10 minutes. pCLE (probe-based confocal laser endomicroscopy) was performed on cell pellets before and 10 minutes after addition of NucView caspase 3 substrate (0.2mM). FIR was calculated and compared between groups using a Kruskall-Wallis’s test. Results are shown as mean and standard deviation (SD) from 5 independent experiments. *p<0.05 (B) & (C) Correlation between pCLE and flow cytometry (B) and between pCLE and epifluorescence microscopy (C).

Eighteen hours after exposure to either saline or 30μg/mL Cisplatin, *in vitro* imaging of A549, H1975, H1650, and HCC827 cell lines by pCLE showed significant differences in FIR between treated and untreated cells (respectively: 2.81±0.32 *vs*. 1.01±0.03, 2.91±0.34 *vs*. 1.01±0.02, 2.45±0.15 *vs*. 1.02±0.01, 3.20±0.32 *vs*. 1.13±0.23; p<0.05). FIR was not different between groups when cells were treated with caspase-3 inhibitor (10μM) (Fig 1A). Moreover, pCLE results were strongly correlated with FACS data (r^2^ = 0.91) (Fig 1B) and with numeration of apoptotic cells using confocal epifluorescence microscopy (r^2^ = 0.92) (Fig 1C). When pCLE was applied on Erlotinib (10μM) treated cells, FIR was significantly different between resistant cells (A549 and H1975) and H1650 insensitive cells (1.06±0.02 and 1.05±0.04 *vs*. 1.94±0.07, p<0.05), between resistant cells and HCC827 hypersensitive cells (1.06±0.02 and 1.05±0.04 *vs*. 3.84±0.43, p<0.05), and between insensitive and hypersensitive cells (1.94±0.07 *vs*. 3.84±0.43, p<0.05).

### *Ex vivo* assessment of Erlotinib sensitivity using apoptosis imaging by pCLE

**Fig 2:**
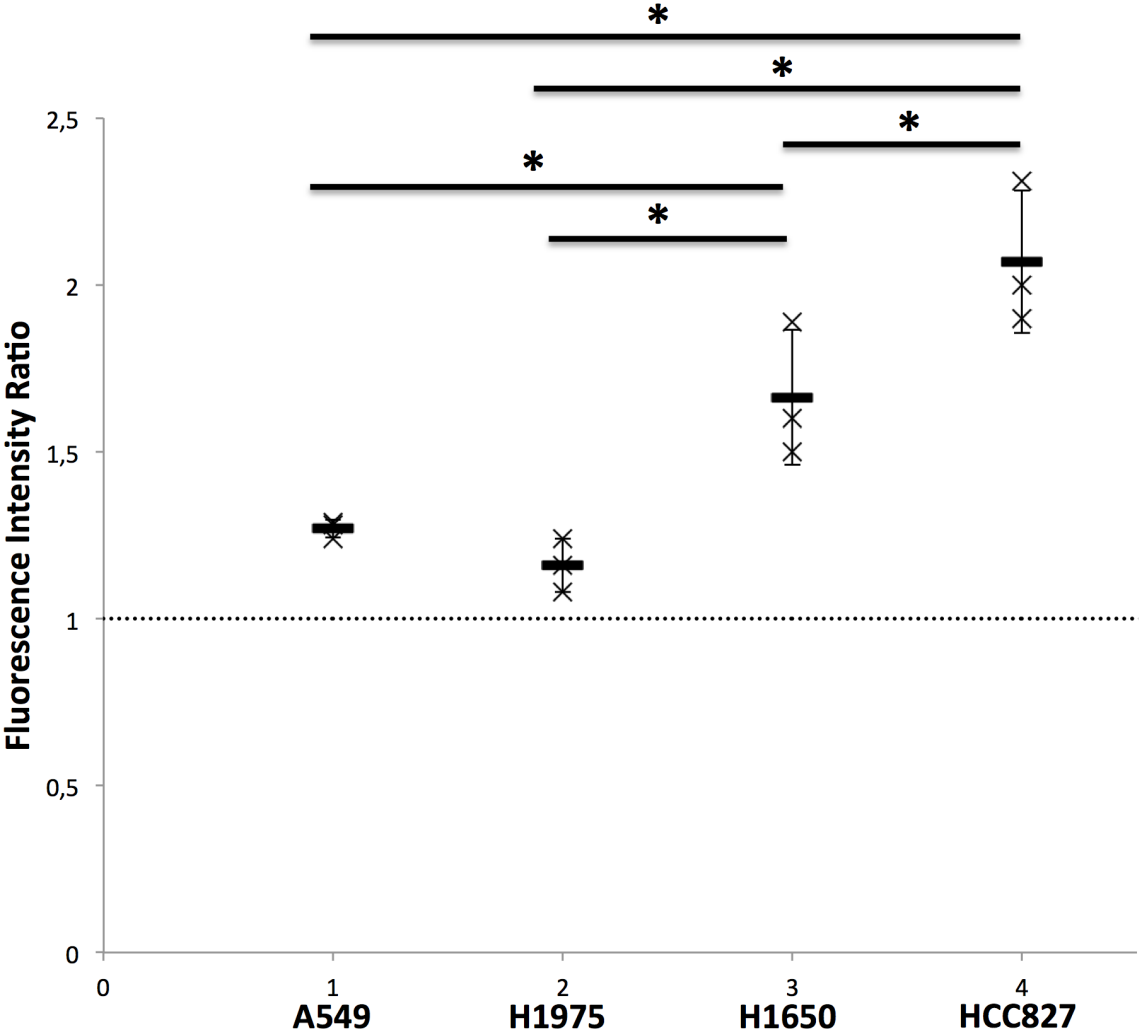
*Ex vivo* assessment of apoptosis using pCLE shows an increased fluorescent signal in erlotinib sensitive tumors. A549, H1975, H1650 and HCC827 tumor xenografts were explanted (experiment performed in triplicate for each cell line) and divided in 3 to 8 fragments. Two to 6 fragments per tumor were treated for 10 minutes with erlotinib (10μM) in 100μL culture medium, 1 fragment per tumor was incubated for 10 minutes with 10% DMSO in 100μL culture medium and 1 fragment per tumor was treated with 100mg/ml Cisplatin in 100μL culture medium. All samples were imaged *ex vivo*. pCLE was performed before and 2 minutes after addition of NucView caspase 3 substrate (1 μM). FIR was calculated and compared between groups using a Kruskall-Wallis’s test. Results are shown as mean and SD from 3 independent experiments. *p<0.05.

Once tumors have reached 125mm^3^, A549, H1975, H1650 and HCC827 tumor xenografts were explanted (n = 3 each), divided in 3 to 8 fragments that were treated for 10 minutes with erlotinib (10ìM) (n = 2 to 6 fragments per tumor), DMSO (n = 1 fragment per tumor), and 10μg Cisplatin (n = 1 fragment per tumor). pCLE was performed before and 2 minutes after addition of NucView caspase 3 substrate (1 μM). The FIR was significantly higher in the HCC827 tumors (2.07±0.21) compared to A549 (1.27±0.03, p<0.05), H1975 (1.16±0.08, p<0.05) and H1650 (1.66±0.20, p<0.05) tumors (Fig 2). The FIR in the insensitive H1650 tumors was also significantly higher when compared to A549 and H1975 (p<0.05) (Fig 2).

### *In vivo* detection of apoptosis using pCLE

**Fig 3:**
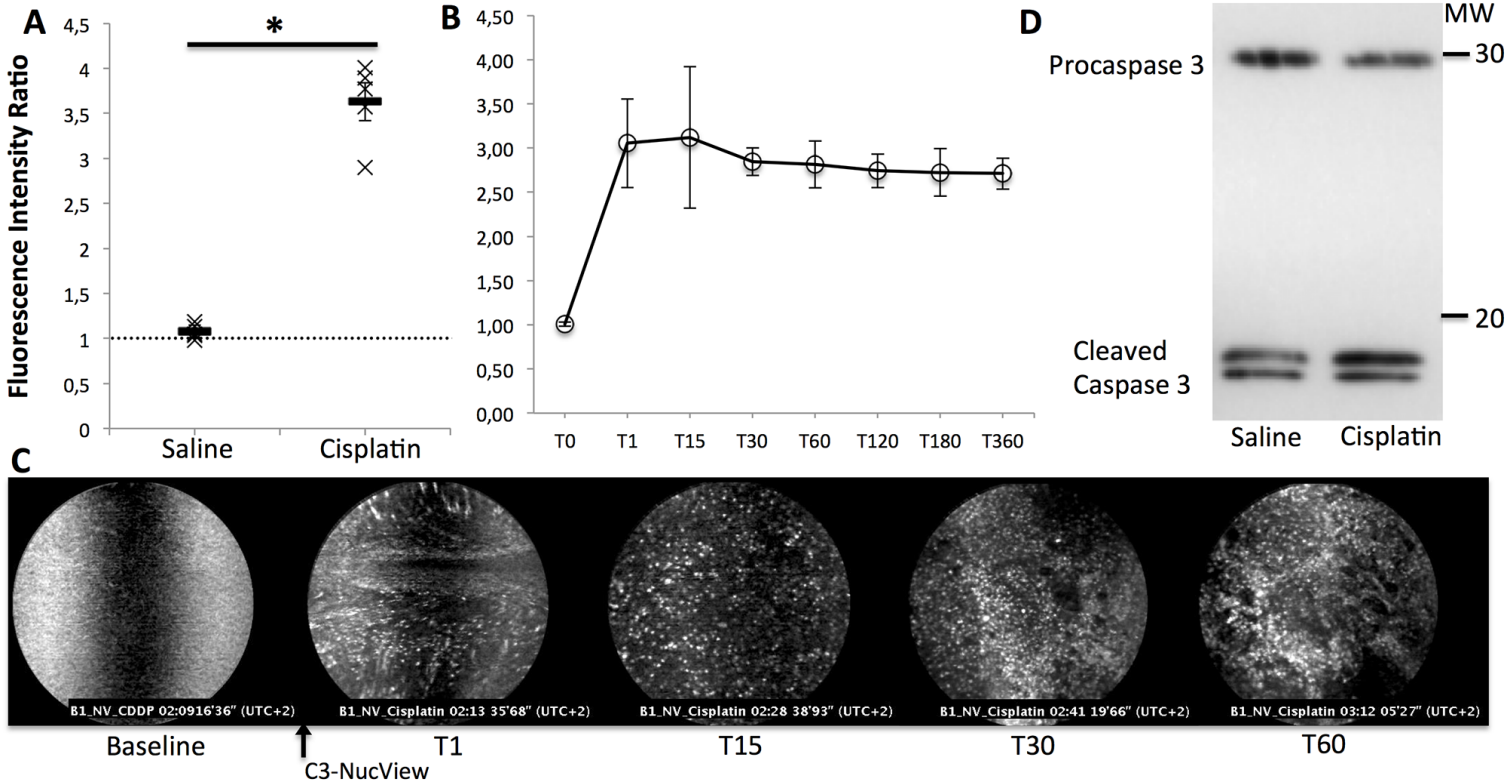
Probe-based confocal laser endomicroscopy enables the *in vivo* detection of apoptosis using C3-NucView in A549 tumor xenografts. “(A) & (B): Five mice with two A549 tumor xenografts per mouse were imaged in vivo 24 hours after treatment with Cisplatin (25μg in 0.05 ml of saline, intra-tumoral injection) or saline (0.05mL, intra-tumoral injection). FIR in A and B were calculated and compared between groups using a Kruskall-Wallis’s test. Results are shown as mean and SD from 5 independent experiments. *p<0.05. (A) pCLE was performed before and 10 minutes after intra-venous infusion of NucView caspase 3 (2nmol). (B) pCLE was performed 1, 15, 30, 60, 120, 180 or 360 minutes after intra-venous infusion of NucView caspase 3 (2nmol). (C) Illustrations of the pCLE *in vivo* imaging of C3-NucView activation in one Cisplatin-treated A549 xenograft, 2 minutes before and 1, 15, 30 and 60 minutes after i.v. injection of 2nmol C3-NucView. (D) Western Blot imaging of activated caspase-3, *ex vivo*, after *in vivo* treatment (intra tumoral injection), in one animal with one tumor treated with cisplatin, and the other one treated with saline, showing a higher level of activated caspase 3 in cisplatin treated tumor.

Twenty-four hours after treatment of A549 xenograft bearing mice by intra-tumoral injection of 50μL saline or 25μg Cisplatin, *in vivo* pCLE imaging of apoptosis showed a higher FIR in treated tumors compared to untreated ones (3.63±0.17 *vs*. 1.03±0.05, p = 0.004) (Fig 3A). These results were consistent with *ex vivo* detection of apoptosis using Western Blot for activated caspase 3 (Fig 3D). The FIR was maximum at one minute after IV injection of the caspase probe and remained stable over up to 360 minutes (Fig 3) on successive imaging procedures of the same tumor.

### *In vivo* assessment of Erlotinib sensitivity using pCLE

**Fig 4:**
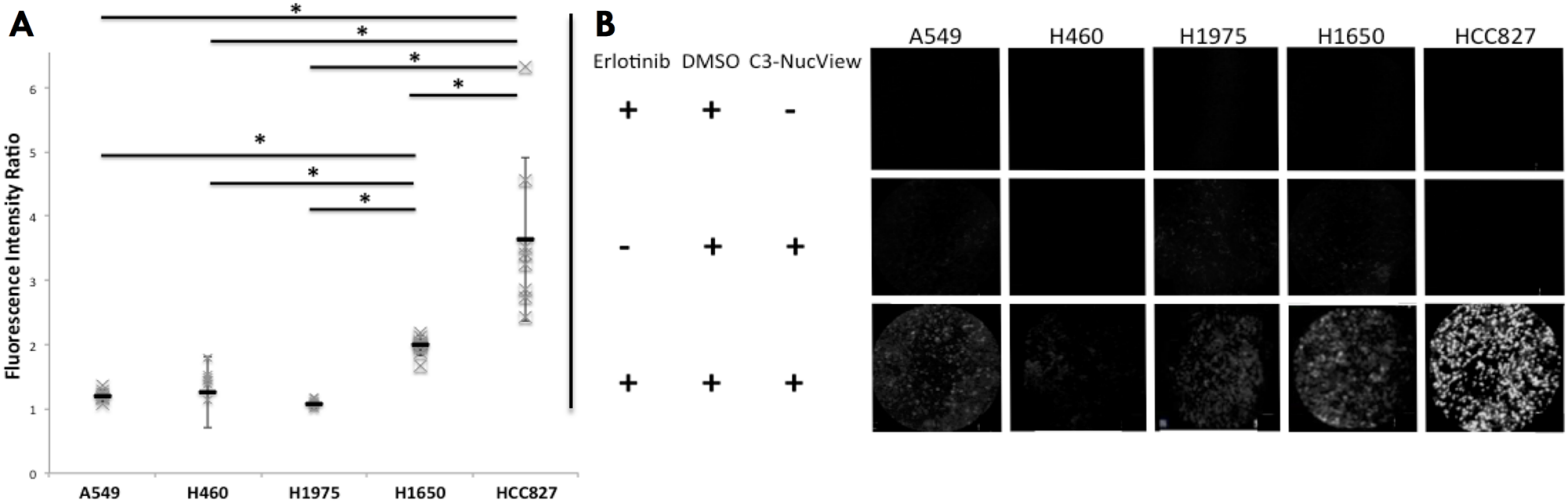
*In vivo* assessment of apoptosis using pCLE differentiates Erlotinib resistant, insensitive and hypersensitive tumors. (A) A549 (n = 8), H460 (n = 7), H1975 (n = 9), H1650 (n = 8) and HCC827 (n = 7) tumor xenografts were imaged *in vivo* 24 hours after treatment with Erlotinib (25mg/kg, intra-peritoneal injection) or DMSO (0.1mL). pCLE was performed before and 10 minutes after intra-venous infusion of NucView caspase 3 inhibitor (2nmol). FIR for DMSO-treated mice was 1.01 (A549), 1.01 (H460), 1.02 (H1975), 1.04 (H1650) and 1.01 (HCC827), (see supplementary material). FIR was compared between groups using a Kruskall-Wallis’s test. Results are shown as mean and SD from independent experiments. *p<0.05. (B) Illustrations of the pCLE *in vivo* imaging of C3-NucView activation in tumor xenografts according to the different cell lines after treatment with erlotinib.

*In vivo* assessment of apoptosis using pCLE in tumor xenografts showed a higher FIR in HCC827 tumors (3.64±1.80) compared to A549 (1.22±0.10, p = 0.01), H460 (1.47±0.27, p = 0.02), H1975 (1.08±0.06, p = 0.01) and H1650 (1.99±0.2, p = 0.01) tumors (Fig 4). The FIR in the H1650 tumors was also higher than in A549, H460 and H1975 tumors (p<0.01, p = 0.03 and p<0.01, respectively) (Fig 4 and S1 table).

## Discussion

Our study shows that an *in vivo in situ* real-time imaging of apoptosis using pCLE enables to differentiate sensitive from resistant tumors to Erlotinib in a mouse model of lung adenocarcinoma, as early as 24 hours after treatment initiation.

Erlotinib induces apoptosis via activation of caspase 3. The pCLE imaging was performed with C3-NucView, a caspase 3 substrate that becomes fluorescent when it reaches the DNA, in the nucleus. In this study, the FIR was correlated to the amount of activated caspase 3 in the cell, and was found to be higher in cells hypersensitive to Erlotinib than in cells less sensitive to Erlotinib. This allowed to differentiate HCC827 tumors from H1650 ones. These two cell lines bear the same *EGFR* activating mutation but a deletion of *PTEN* lowers the efficacy of Erlotinib in the H1650 cells [34]. This particular situation illustrates a major interest of *in vivo* molecular imaging over genetic analysis of *EGFR*: providing a comprehensive phenotypic information in the individual context of a patient. In fact, *in vivo* imaging of the response to therapeutic takes into account every potential resistance mechanism, including, in the case of Erlotinib, other genomic abnormalities such as *EGFR* amplification, polymorphism or over-expression, *PTEN* loss or *BIM* polymorphism and pharmacodynamic variations [35]. A limitation of our study is the number of cell lines used for this proof of concept study. According to our results, increasing the panel of sensitive / resistant cell line may allow to better distinguish different profiles of sensitivity to erlotinib.

The in vivo technique developed here can be used for the early assessment of tumor response to Erlotinib. Nevertheless, if pCLE is available in the clinical setting, the fluorescent probe used in this study is not. Therefore, we developed an ex vivo test using the same technique, in incubating fresh biopsies consecutively with EGFR TKI and caspase probe ex-vivo. The cumulative incubation time of Erlotinib and Caspase probe was limited to 15 minutes including 2 minutes in the caspase probe mix, in order to be compatible with a rapid on site procedure that would be conducted in an endoscopic suite immediately after a bronchial biopsy.

Here again, hypersensitive tumors could be differentiated from insensitive and from resistant ones. The use of pCLE allows its implementation in the bronchoscopy suite, as a rapid on-site *ex vivo* test. However, such a test would require to control the tumoral content of the evaluated biopsy. To address this particular issue, biopsy guidance using endo-bronchial ultrasound and rapid on-site evaluation of the biopsy by a pathologist can be proposed.

A potential pitfall of pCLE is the influence of the cellular density on the fluorescent signal. To limit this bias, we set a 100 UA lower limit for the Look Up Table during image analysis. This setting allows to reduce the background noise and excludes non fluorescent areas of the analysis. Thus, the FIR reflects the median fluorescence intensity of the nuclei if there are apoptotic cells in the field of view, and is close to 1 if not. Eventually, in the hypothetical case of a high FIR with very few nuclei, the observer would eliminate the corresponding images from the analysis in order to avoid the quantitation of irrelevant signal.

## Conclusions

This study shows that micro-imaging of apoptosis using pCLE with C3-NucView enables the differentiation of hypersensitive, insensitive and resistant tumors to Erlotinib, both *in vivo* and *ex vivo*. Although the technique was applied to Erlotinib sensitivity assessment only, it can be anticipated that it can be used for other drugs that induce apoptosis. If developed for *ex-vivo* analysis of patients’ samples, giving the growing number of targeted therapies in NSCLC and other tumors -with different resistance mechanisms for several of them-, a unique assessment method could be preferred to multiple genetic analysis, as it limits implementation costs and allows immediate testing for new molecules.

## Supporting information

S1 Table FIR from in vitro, ex vivo and in vivo pCLE assessment of apoptosis using C3-NucView. FIR is given for experiments using C3-NucView pCLE of A549, H460, H1975, H1650 and HCC827 cell lines (*in vitro*) and tumor xenografts (*ex vivo* and *in vivo*). VEH: vehicle. CDDP: cisplatin. INH: caspase 3 inhibitor. ERLO: Erlotinib.
(XLSX)
